# Digital Tools to Ameliorate Psychological Symptoms Associated With COVID-19: Scoping Review

**DOI:** 10.2196/19706

**Published:** 2020-08-21

**Authors:** Melvyn Zhang, Helen Elizabeth Smith

**Affiliations:** 1 Family Medicine and Primary Care Lee Kong Chian School of Medicine Nanyang Technological University Singapore Singapore

**Keywords:** COVID-19, digital tool, psychiatry, mental health, digital health, psychology, distress, stress, anxiety, depression

## Abstract

**Background:**

In the four months after the discovery of the index case of coronavirus disease (COVID-19), several studies highlighted the psychological impact of COVID-19 on frontline health care workers and on members of the general public. It is evident from these studies that individuals experienced elevated levels of anxiety and depression in the acute phase, when they first became aware of the pandemic, and that the psychological distress persisted into subsequent weeks. It is becoming apparent that technological tools such as SMS text messages, web-based interventions, mobile interventions, and conversational agents can help ameliorate psychological distress in the workplace and in society. To our knowledge, there are few publications describing how digital tools have been used to ameliorate psychological symptoms among individuals.

**Objective:**

The aim of this review was to identify existing SMS text message, web-based, mobile, and conversational agents that the general public can access to ameliorate the psychological symptoms they are experiencing during the COVID-19 pandemic.

**Methods:**

To identify digital tools that were published specifically for COVID-19, a search was performed in the PubMed and MEDLINE databases from the inception of the databases through June 17, 2020. The following search strings were used: “NCOV OR 2019-nCoV OR SARS-CoV-2 OR Coronavirus OR COVID19 OR COVID” and “mHealth OR eHealth OR text”. Another search was conducted in PubMed and MEDLINE to identify existing digital tools for depression and anxiety disorders. A web-based search engine (Google) was used to identify if the cited web-based interventions could be accessed. A mobile app search engine, App Annie, was used to determine if the identified mobile apps were commercially available. Results: A total of 6 studies were identified. Of the 6 identified web-based interventions, 5 websites (83%) could be accessed. Of the 32 identified mobile interventions, 7 apps (22%) could be accessed. Of the 7 identified conversational agents, only 2 (29%) could be accessed.

**Results:**

A total of 6 studies were identified. Of the 6 identified web-based interventions, 5 websites (83%) could be accessed. Of the 32 identified mobile interventions, 7 apps (22%) could be accessed. Of the 7 identified conversational agents, only 2 (29%) could be accessed.

**Conclusions:**

The COVID-19 pandemic has caused significant psychological distress. Digital tools that are commercially available may be useful for at-risk individuals or individuals with pre-existing psychiatric symptoms.

## Introduction

On December 31, 2019, the World Health Organization (WHO) was alerted to a case of pneumonia of unknown cause that originated from China [1]. The causative pathogen was a novel coronavirus, severe acute respiratory syndrome coronavirus 2 (SARS-CoV-2), and the disease it causes is now referred to as coronavirus disease (COVID-19) [1]. As numerous other individuals became afflicted, the WHO declared the outbreak of COVID-19 to be an international public health emergency [1]. The problem escalated, with many individuals across different countries being afflicted; on March 11, 2020, the WHO increased their alert to a pandemic [1]. The rapid escalation of the number of individuals infected, the increasing number of deaths, and the measures undertaken by governments have resulted in significant psychological impact not only on health care workers, but also on the public [2].

In the four months after the discovery of the index case, several studies highlighted the psychological impact of COVID-19 on frontline health care workers and on members of the public. Li et al [3] used a mobile phone app to administer the Chinese version of a vicarious traumatization questionnaire to 214 individuals from the public and 526 nurses (234 frontline nurses and 292 nurses not on the front line). They found that the vicarious traumatization scores were higher among the public and nonfrontline health care workers. Wang et al [4] investigated the mental health status of 1120 members of the public living in China using both the Impact of Event Scale–Revised (IES-R) and the Depression, Anxiety, and Stress Scale (DASS-21). Notably, 53.8% of respondents reported the psychological impact to be moderate to severe, with 16.5% having moderate to severe depressive symptoms, 28.8% having moderate to severe anxiety symptoms, and 8.1% having moderate to severe stress. These studies focused on the immediate psychological impact of the COVID-19 pandemic; meanwhile, in their most recent study, Wang et al [5] examined the longitudinal changes in the mental health of the general population in China. A total of 1738 participants were included, and they were administered the IES-R questionnaire and the DASS-21 at baseline and after 4 weeks. While there was a mean reduction in the overall scores across a period of 4 weeks, the mean scores for the IES-R scale were still high, suggesting the presence of posttraumatic stress disorder (PTSD) symptoms [5]. It is evident from these studies that individuals experienced elevated levels of anxiety and depression in the acute phase, when they first became aware of the pandemic, and that this psychological distress persisted into subsequent weeks.

Since the onset of the pandemic, the priorities for government have understandably been the treatment of people infected with COVID-19 and steps to limit spread. It is evident in many countries that the need for clinical services has exceeded the supply, requiring the construction of temporary medical facilities and redeployment of staff. Beyond hospitals, the impact of COVID-19 is multifactorial; economies are being affected, individuals are suffering bereavement after loss of loved ones, and others are physically isolated and quarantined. Social distancing and lockdowns have created difficulties in accessing mental health services. Individuals with psychiatric disorders are likely to have increasing difficulty accessing conventional mental health services. Hao et al [6] reported that mean PTSD, anxiety, depression, and insomnia scores were elevated in psychiatric patients compared to those in the general population. A need indeed exists for web-based mental health interventions that use digital tools. An editorial has also been published describing how web-based tools and social media have been used in China to support the mental health needs of frontline workers as well as of people who are infected or living in quarantine facilities [7]. It is becoming apparent that technological tools such as SMS text messages, web-based interventions, mobile interventions, and conversational agents can help ameliorate psychological distress in the workplace and society. In an opinion paper, Zhou et al [8] described the efforts of the Australian government to provide telemental health solutions to address the psychological impact of COVID-19. Unfortunately, the scope of this paper was limited to the identification of relevant services in Australia. In another recent paper, Cosic et al [9] highlighted the potential of digital tools in dealing with the psychological distress associated with COVID-19 and how their prior experiences can be applied in developing relevant apps; however, the authors failed to identify any existing digital tools that individuals can use. To our knowledge, except for the paper by Zhou et al [8], few publications have described how digital tools are being used to ameliorate psychological symptoms among individuals. Thus, our aim in this paper was to identify existing SMS text message, web-based, mobile, and conversational agents that the public can access to ameliorate the psychological symptoms they are facing during the COVID-19 pandemic.

## Methods

To identify digital tools that have been published specifically for COVID-19, a search was performed through PubMed and MEDLINE from the inception of the databases through June 17, 2020. The following search strings were used: “*NCOV* OR *2019-nCoV* OR *SARS-CoV-2* OR *Coronavirus* OR *COVID19* OR *COVID*” and “*mHealth* OR *eHealth* OR *text*”. Given that the aim was to identify potential digital tools, the terms electronic health (*eHealth*), mobile health (*mHealth*), and *text* were used because these terminologies would identify all potential web-based, mobile, and SMS text message interventions.

Another search was conducted on PubMed and MEDLINE to identify existing digital tools for depression and anxiety disorders. To identify these tools, reviews of digital tools (SMS text messaging, web-based interventions, mobile apps, and conversational agents) were identified. Only articles in the English language were considered. A narrative synthesis of the identified tools was conducted.

A web-based search engine (Google) was used to identify if the cited web-based interventions could be accessed. A mobile app search engine, App Annie [10], was used to identify if the mobile apps identified in the literature search were commercially available. All available data have been included in the manuscript.

## Results

Based on the search strategy, a total of 9829 articles were identified from the databases. Of these, 80/9829 (0.8%) were duplicated references. Upon further screening, a total of 24/9829 articles (0.2%) were identified as potentially relevant to COVID-19. Upon further examination of the full texts of these 24 articles, only one article described how an SMS text messaging intervention was applied to address mental health issues resulting from COVID-19 [11]. The remaining articles did not describe or mention how the discussed SMS text message, web-based, or mobile interventions helped ameliorate the psychological symptoms associated with COVID-19.

In our search for existing digital tools to manage depression and anxiety disorders, a total of 5 articles were identified. Two web-based reviews were identified for depressive disorders, and another was identified for anxiety disorders. Of the identified web-based interventions, we managed to access 5 of the 6 listed websites (83%), namely Beating the Blues [12], Living Life to the Full [13], Deprexis [14], moodgym [15], and Interapy [16]. One review of mobile interventions for depressive and anxiety disorders was identified. Of the identified mobile interventions, only 7/32 apps (22%) were available in commercial stores, namely Angesthjalpen, AnxietyCoach, SmartCAT, Headgear, MoodHacker, SuperBetter, and Thought Challenger. Another review highlighted conversational agents for psychiatric disorders. Of the 7 identified conversational agents, only 2 (29%) were commercially available. Table 1 provides a summarized overview of the identified studies.

**Table 1:** Overview of the studies identified in the literature search.

Study	Year	Mechanism of delivery of digital tools	Identified digital tools and prior evaluations	Availability
Agyapong et al [11]	2020	SMS text messaging (specific to COVID-19^a^)	Text4Hope (specific to COVID-19) enables subscribers to receive 3 months of daily supportive SMS text messages with or without web links to web-based mental health resources.	Only available to individuals living in Alberta, Canada.
Rodriguez-Pulido et al [17]	2020	Web-based Interventions	Beating the Blues is a web-based intervention for depressive disorder.	Available to users in the United Kingdom.
Burger et al [18]	2020	Web-based interventions	Living Life to the Full (2 comparative trials were performed involving a total of 659 participants), Deprexis (6 comparative trials were performed involving a total of 1863 participants), and SHADE (3 comparative trials performed involving a total of 475 participants) were evaluated. moodgym was extensively evaluated, with a total of 11 comparative trials involving a total of 7294 participants. All the above interventions have been evaluated as websites that provide psychological therapy for depressive disorders.	The websites for Living Life to the Full, Deprexis, and moodgym can be accessed.
Anderson et al [19]	2019	Web-based interventions	The Interapy program from the Netherlands was highlighted as a program that assisted individuals with symptoms of depression, panic disorder, posttraumatic stress disorder, and burnout. moodgym was also highlighted as a commercially available option for anxiety and depression.	The websites for moodgym and Interapy can be accessed.
Miralles et al [20]	2020	Mobile interventions	7 Cups, Be Good to Yourself, Bluewatch, Dcombat, Get Happy Program, Headgear, iCare Prevent, MedLink, Mobile Sensing and Support, Moodhacker, Moodivate, MyGamePlan, PRIME-D, Push-D, SocioEmpathy, SPSRS, SuperBetter, The Sound Advice, Thought Challenger, TODAC, Kokoro-App, Agoraphobia free, Stress Free, Angesthjalpen, AnxietyCoach, CBT Assistant, Challenger, Lantern, Psych Assist, Public Speech Trainer, SmartCAT, and GET.ON.PAPP have been previously evaluated and reported in published research.	Headgear, MoodHacker, SuperBetter, Thought Challenger, Angesthjalpen, AnxietyCoach, and SmartCAT are commercially available.
Gaffney et al [21]	2019	Conversational agents or chatbots	Woebot, Tess, and eSMART-TH have been evaluated previously for depressive disorder, SABORI has been evaluated for psychological distress, and Tess has been evaluated for anxiety disorder.	Woebot and Tess are commercially available.

^a^COVID-19: coronavirus disease.

## Discussion

### Principal Findings

This review is one of the first to examine the literature for digital interventions that can be used by the general public as well as specific groups, such as workers and health care professionals, to ameliorate the psychological distress they are experiencing during the COVID-19 pandemic as well as specific symptoms such as panic buying [22-25]. The findings from our paper complement those of Zhou et al [8], who highlighted tools in their article that can be accessed by individuals in Australia. Our review helped address some of the inherent limitations of the work by Zhou et al [8], given that the authors only listed available resources without providing any evidence-based justification of the suggested interventions. We identified an SMS text message–based intervention that was designed to address the mental health needs of individuals in Canada. We managed to identify web-based, mobile, and conversational agents that are commercially available and have been previously validated by research.

Our review only identified one publication that describes how SMS text messaging technologies are used as a form of psychological support. As mentioned in the Introduction, numerous studies have been published that characterize the immediate and delayed psychological impact of COVID-19 on medical workers and members of the public [2,4,5]. There is still a lack of evaluation of psychological tools to address the identified psychological concerns, namely heightened levels of stress, anxiety, and depression. Psychological distress occurs frequently in everyday life; however, during epidemics and pandemics such as COVID-19, the prevalence of distress is extremely high and existing mental health services are unable to function normally. This provides strong justification for the rapid identification of tools that have an evidence base and can be promoted rapidly to address the unmet psychological needs of individuals. Our research highlighted commercially available digital tools (web-based, mobile, and conversational agents) that individuals can access during the COVID-19 pandemic. It is challenging for members of the public to identify tools that have been proven to be clinically effective and are available commercially; hence, our review is important. We focused primarily on reviews of digital tools to identify such tools. Our methods helped address the limitations of the prior work by Zhou et al [8], as they suggested tools but made no attempt to review the evidence base of those tools. The evidence-based websites, smartphone apps, and conversational agents we identified can help ameliorate symptoms of depression and anxiety. These tools have wide application; they can help individuals who are at risk of developing an illness or help individuals with pre-existing illness to cope with these symptoms. This is important because it is anticipated that it will be challenging for people to obtain appropriate psychiatric care as governments impose lockdowns and curb movement.

We identified a variety of commercially available tools; however, there may be limitations to some of these tools. Validations may have been conducted in certain localities or regions, and we cannot be sure that these tools will be as effective in other localities. However, we propose that a tool that has undergone validation, if only in a precise locality, is likely to be superior to a tool without any validation. We also recognize that accessing smartphone apps may be difficult in a different region or country.

It is evident from this paper that psychological tools to help individuals cope with heightened stress, anxiety, and depression due to COVID-19 are lacking. While some commercial tools are available, they are not without limitations. It is important for academic researchers, clinicians, and developers to work jointly to conceptualize tools that can be used by the general population to ameliorate their symptoms of psychological distress. It may also be valuable to consider participatory action research design when conceptualizing new tools to ensure that the created tools better meet the needs of individuals. It may be wise to consider modification of existing tools so that versatile tools can be rapidly deployed to meet the increasing need. In the interim period, while comprehensive treatment tools may not yet be available, we learned from the editorial by Liu et al [7] that in China, helplines and social media platforms are being used to extend support to individuals who are experiencing psychological distress. Similarly, in other countries such as Singapore, the government has set up a mental health hotline to address the needs of the public and to refer at-risk individuals to appropriate mental health services. These clinical services provide some form of supportive therapy; however, there is still a need for tools that provide individuals with more comprehensive treatment, such as cognitive behavioral therapy for depression or anxiety.

### Strengths and Limitations

The strength of this paper is that we examined the literature for digital tools that have been validated, which can help with depressive and anxiety symptoms. In addition, we conducted a search to determine if these tools were available commercially. It is possible, despite these strengths, that we have missed some tools; to mitigate this, we included recently published reviews, but we acknowledge that their search for digital interventions may not be as recent.

### Conclusions

The COVID-19 pandemic has caused significant psychological distress. Commercially available digital tools may be useful for at-risk individuals or individuals with pre-existing psychiatric symptoms. The tools we identified may help address the psychological distress individuals are experiencing during the COVID-19 pandemic.

## Abbreviations

COVID-19: coronavirus disease
DASS-21: Depression, Anxiety, and Stress Scale
eHealth: electronic health
IES-R: Impact of Event Scale–Revised
mHealth: mobile health
PTSD: posttraumatic stress disorder
SARS-CoV-2: severe acute respiratory syndrome coronavirus 2
WHO: World Health Organization

## References

[1] Coronavirus disease (COVID-19) pandemic. World Health Organization.

[2] Rajkumar RP (2020). COVID-19 and mental health: A review of the existing literature. Asian J Psychiatr.

[3] Li Z, Ge J, Yang M, Feng J, Qiao M, Jiang R, Bi J, Zhan G, Xu X, Wang L, Zhou Q, Zhou C, Pan Y, Liu S, Zhang H, Yang J, Zhu B, Hu Y, Hashimoto K, Jia Y, Wang H, Wang R, Liu C, Yang C (2020). Vicarious traumatization in the general public, members, and non-members of medical teams aiding in COVID-19 control. Brain Behav Immun.

[4] Wang C, Pan R, Wan X, Tan Y, Xu L, Ho CS, Ho RC (2020). Immediate Psychological Responses and Associated Factors during the Initial Stage of the 2019 Coronavirus Disease (COVID-19) Epidemic among the General Population in China. Int J Environ Res Public Health.

[5] Wang C, Pan R, Wan X, Tan Y, Xu L, McIntyre RS, Choo FN, Tran B, Ho R, Sharma VK, Ho C (2020). A longitudinal study on the mental health of general population during the COVID-19 epidemic in China. Brain Behav Immun.

[6] Hao F, Tan W, Jiang L, Zhang L, Zhao X, Zou Y, Hu Y, Luo X, Jiang X, McIntyre RS, Tran B, Sun J, Zhang Z, Ho R, Ho C, Tam W (2020). Do psychiatric patients experience more psychiatric symptoms during COVID-19 pandemic and lockdown? A case-control study with service and research implications for immunopsychiatry. Brain Behav Immun.

[7] Liu S, Yang L, Zhang C, Xiang Y, Liu Z, Hu S, Zhang B (2020). Online mental health services in China during the COVID-19 outbreak. Lancet Psychiat.

[8] Zhou X, Snoswell CL, Harding LE, Bambling M, Edirippulige S, Bai X, Smith AC (2020). The Role of Telehealth in Reducing the Mental Health Burden from COVID-19. Telemed J E Health.

[9] Ćosić K, Popović S, Šarlija M, Kesedžić I (2020). Impact of Human Disasters and COVID-19 Pandemic on Mental Health: Potential of Digital Psychiatry. Psychiatr Danub.

[10] App Annie.

[11] Agyapong VI (2020). Coronavirus Disease 2019 Pandemic: Health System and Community Response to a Text Message (Text4Hope) Program Supporting Mental Health in Alberta. Disaster Med Public Health Prep.

[12] Beating the Blues.

[13] Living Life to the Full.

[14] Deprexis.

[15] moodgym.

[16] Interapy. Webpage in Dutch.

[17] Rodriguez-Pulido F, Castillo G, Hamrioui S, Martin LD, Vazquez-Beltrán P, de la Torre-Díez I, Franco-Martín MA (2020). Treatment of Depression in Primary Care with Computerized Psychological Therapies: Systematic Reviews. J Med Syst.

[18] Burger F, Neerincx MA, Brinkman W (2020). Technological State of the Art of Electronic Mental Health Interventions for Major Depressive Disorder: Systematic Literature Review. J Med Internet Res.

[19] Andersson G, Carlbring P, Titov N, Lindefors N (2019). Internet Interventions for Adults with Anxiety and Mood Disorders: A Narrative Umbrella Review of Recent Meta-Analyses. Can J Psychiatry.

[20] Miralles I, Granell C, Díaz-Sanahuja L, Van Woensel W, Bretón-López J, Mira A, Castilla D, Casteleyn S (2020). Smartphone Apps for the Treatment of Mental Disorders: Systematic Review. JMIR mHealth uHealth.

[21] Gaffney H, Mansell W, Tai S (2019). Conversational Agents in the Treatment of Mental Health Problems: Mixed-Method Systematic Review. JMIR Ment Health.

[22] Tan W, Hao F, McIntyre RS, Jiang L, Jiang X, Zhang L, Zhao X, Zou Y, Hu Y, Luo X, Zhang Z, Lai A, Ho R, Tran B, Ho C, Tam W (2020). Is returning to work during the COVID-19 pandemic stressful? A study on immediate mental health status and psychoneuroimmunity prevention measures of Chinese workforce. Brain Behav Immun.

[23] Chew NW, Lee GK, Tan BY, Jing M, Goh Y, Ngiam NJ, Yeo LL, Ahmad A, Ahmed Khan F, Napolean Shanmugam G, Sharma AK, Komalkumar R, Meenakshi P, Shah K, Patel B, Chan BP, Sunny S, Chandra B, Ong JJ, Paliwal PR, Wong LY, Sagayanathan R, Chen JT, Ying Ng AY, Teoh HL, Tsivgoulis G, Ho CS, Ho RC, Sharma VK (2020). A multinational, multicentre study on the psychological outcomes and associated physical symptoms amongst healthcare workers during COVID-19 outbreak. Brain Behav Immun.

[24] Tan BY, Chew NW, Lee GK, Jing M, Goh Y, Yeo LL, Zhang K, Chin H, Ahmad A, Khan FA, Shanmugam GN, Chan BP, Sunny S, Chandra B, Ong JJ, Paliwal PR, Wong LY, Sagayanathan R, Chen JT, Ying Ng AY, Teoh HL, Ho CS, Ho RC, Sharma VK (2020). Psychological Impact of the COVID-19 Pandemic on Health Care Workers in Singapore. Ann Intern Med.

[25] Ho C, Chee C, Ho R (2020). Mental Health Strategies to Combat the Psychological Impact of COVID-19 Beyond Paranoia and Panic. Ann Acad Med Singapore.

